# In Vitro Seeding Activity of Glycoform-Deficient Prions from Variably Protease-Sensitive Prionopathy and Familial CJD Associated with PrP^V180I^ Mutation

**DOI:** 10.1007/s12035-018-1459-0

**Published:** 2019-01-05

**Authors:** Zerui Wang, Jue Yuan, Pingping Shen, Romany Abskharon, Yue Lang, Johnny Dang, Alise Adornato, Ling Xu, Jiafeng Chen, Jiachun Feng, Mohammed Moudjou, Tetsuyuki Kitamoto, Hyoung-gon Lee, Yong-Sun Kim, Jan Langeveld, Brian Appleby, Jiyan Ma, Qingzhong Kong, Robert B. Petersen, Wen-Quan Zou, Li Cui

**Affiliations:** 1 Department of Neurology, The First Hospital of Jilin University, Changchun, Jilin Province People’s Republic of China; 2 Department of Pathology, Case Western Reserve University School of Medicine, Cleveland, OH USA; 3 Center for Neurodegenerative Science, Van Andel Research Institute, Grand Rapids, MI 49503 USA; 4 Virologie Immunologie Moléculaires, INRA, Jouy-en-Josas, France; 5 Center for Prion Diseases, Tohoku University Graduate School of Medicine, 2-1 Seiryo-machi, Aoba-ku, Sendai, 980-8575 Japan; 6 Department of Biology, College of Sciences, University of Texas at San Antonio, San Antonio, TX 78249 USA; 7 Department of Microbiology, College of Medicine, Hallym University, Chuncheon, Gangwon-do 24252 Republic of Korea; 8 Wageningen BioVeterinary Research, Houtribweg 39, Lelystad, the Netherlands; 9 National Prion Disease Pathology Surveillance Center, Case Western Reserve University School of Medicine, Cleveland, OH USA; 10 Department of Neurology, Case Western Reserve University School of Medicine, Cleveland, OH USA; 11 Foundation Sciences, Central Michigan University College of Medicine, Mount Pleasant, MI USA; 12 State Key Laboratory for Infectious Disease Prevention and Control, National Institute for Viral Disease Control and Prevention, China Center for Disease Control and Prevention, Beijing, China

**Keywords:** Prion, Prion disease, Variably protease-sensitive prionopathy (VPSPr), Serial protein misfolding cyclic amplification (sPMCA), Real-time quaking-induced conversion (RT-QuIC), Humanized transgenic mice, Polymorphism

## Abstract

Both sporadic variably protease-sensitive prionopathy (VPSPr) and familial Creutzfeldt-Jakob disease linked to the prion protein (PrP) V180I mutation (fCJD^V180I^) have been found to share a unique pathological prion protein (PrP^Sc^) that lacks the protease-resistant PrP^Sc^ glycosylated at residue 181 because two of four PrP glycoforms are apparently not converted into the PrP^Sc^ from their cellular PrP (PrP^C^). To investigate the seeding activity of these unique PrP^Sc^ molecules, we conducted in vitro prion conversion experiments using serial protein misfolding cyclic amplification (sPMCA) and real-time quaking-induced conversion (RT-QuIC) assays with different PrP^C^ substrates. We observed that the seeding of PrP^Sc^ from VPSPr or fCJD^V180I^ in the sPMCA reaction containing normal human or humanized transgenic (Tg) mouse brain homogenates generated PrP^Sc^ molecules that unexpectedly exhibited a dominant diglycosylated PrP isoform along with PrP monoglycosylated at residue 181. The efficiency of PrP^Sc^ amplification was significantly higher in non-CJDMM than in non-CJDVV human brain homogenate, whereas it was higher in normal TgVV than in TgMM mouse brain homogenate. PrP^C^ from the mixture of normal TgMM and Tg mouse brain expressing PrP^V180I^ mutation (Tg180) but not TgV180I alone was converted into PrP^Sc^ by seeding with the VPSPr or fCJD^V180I^. The RT-QuIC seeding activity of PrP^Sc^ from VPSPr and fCJD^V180I^ was significantly lower than that of sCJD. Our results suggest that the formation of glycoform-selective prions may be associated with an unidentified factor in the affected brain and the glycoform-deficiency of PrP^Sc^ does not affect the glycoforms of in vitro newly amplified PrP^Sc^.

**Electronic supplementary material:**

The online version of this article (10.1007/s12035-018-1459-0) contains supplementary material, which is available to authorized users.

## Introduction

Prions are infectious pathogens that are associated with a group of fatal transmissible spongiform encephalopathies or prion diseases affecting both animals and humans. They are composed mainly of the pathologic scrapie conformers (PrP^Sc^) that originate from their normal cellular prion protein (PrP^C^) through a conformational transition of the largely α-helical form to predominantly β-sheets by a seed- or template-assisted mechanism [1, 2]. Human prion diseases are highly heterogeneous: they can be familial, sporadic, or acquired by infection, and include Creutzfeldt-Jakob disease (CJD), Gerstmann-Sträussler-Scheinker (GSS) disease, fatal insomnia, kuru, variant CJD (vCJD), and variably protease-sensitive prionopathy (VPSPr) [3].

PrP^Sc^ detected in the brain of all sporadic and acquired CJD and in most familial CJD has been well recognized to be mainly composed of three typical PrP glycoforms including diglycosylated, monoglycosylated, and un-glycosylated forms. However, Zou and co-workers recently discovered that the sporadic VPSPr along with familial Creutzfeldt-Jakob disease (fCJD) associated with a PrP mutation Val to Ile at residue 180 (fCJD^V180I^) generates a PrP^Sc^ molecule with a unique electrophoretic gel profile and is characterized by the accumulation in the brain of PrP^Sc^ that lacks diglycosylated PrP^Sc^ and PrP^Sc^ monoglycosylated at residue 181 [4–6]. Unlike fCJD linked to the PrP mutation Thr to Ala at residue 183 (fCJD^T183A^) that eliminates the *N*-linked glycosylation at residue 181 [7–9], the overall cellular PrP from VPSPr and fCJD^V180I^ shows a normal PrP profile with the highest amount being diglycosylated PrP, followed by monoglycosylated PrP, and the lowest amount being un-glycosylated PrP prior to digestion with proteinase K (PK). Therefore, at least based on Western blotting, PrP from VPSPr and fCJD^V180I^ seems to have an intact glycoform profile similar to that from normal controls but somehow the diglycosylated PrP and PrP monoglycosylated at residue 181 are not converted into the PK-resistant PrP^Sc^; only un-glycosylated PrP and PrP monoglycosylated at residue 197 are converted into PK-resistant PrP^Sc^ [4–6]. Interestingly, two subsequent studies revealed that PrP^Sc^ from VPSPr patients exhibited very low transmissibility in the first passage of a transmission study in humanized transgenic mice and no transmission was observed at all in the second passage [10, 11].

To further understand the molecular mechanism underlying the conversion of PrP^C^ into this unique PrP^Sc^ in VPSPr and fCJD^V180I^, we employed two advanced technologies termed serial protein misfolding cyclic amplification (sPMCA) and real-time quaking-induced conversion (RT-QuIC) assays to investigate the in vitro seeding activity of PrP^Sc^ from patients with these two conditions, respectively. We found that PrP^Sc^ from VPSPr and fCJD^V180I^ can be amplified very efficiently using the brain homogenate from non-CJD individuals carrying PrP-129MM but poorly using the brain homogenate from non-CJD individuals carrying PrP-129VV. With PrP substrates from humanized transgenic mice expressing human wild-type PrP-129MM (TgMM) or PrP-129VV (TgVV) or expressing human mutant PrP with V180I mutation coupled with PrP-129MM polymorphism (TgV180I), amplification of PrP^Sc^ from VPSPr and fCJD^V180I^ was highly efficient using TgVV and poor using TgMM or TgV180I alone. Interestingly, PrP^Sc^ was very efficiently amplified using a mixture of brain homogenate derived from both TgMM and TgV180I. As a control, we found that PrP^Sc^ from fCJD^T183A^ that lacks the *N*-linked glycosylation at residue 181 could be amplified in non-CJD human brain homogenate with either PrP-129MM or PrP-129VV. PrP^Sc^ from fCJD^T183A^ was also very efficiently amplified using brain homogenates from TgMM and TgMM + TgV180I, but not amplified using TgVV or TgV180I alone. Surprisingly, diglycosylated PrP^Sc^ was the predominant form in all amplified PrP^Sc^ species. Moreover, RT-QuIC assay demonstrated that PrP^Sc^ from VPSPr, fCJD^V180I^, and fCJD^T183A^ was all amplifiable using recombinant bank vole PrP as a substrate; the seeding activity of PrP^Sc^ from VPSPr and fCJD^V180I^ was approximately 10^2^- to 10^5^-fold lower than that of PrP^Sc^ from sCJDMM1 and sCJDVV2.

## Materials and Methods

### Reagents and Antibodies

PK was purchased from Sigma Chemical Co. (St. Louis, MO). Reagents for enhanced chemiluminescence (ECL Plus) were from Amersham Pharmacia Biotech, Inc. (Piscataway, NJ). Anti-PrP antibodies 3F4 against human PrP residues 107-112 [12, 13], 1E4 against human PrP97-106 [14], Tohoku 2 (T2) [15], Bar209, and V14 [4, 16] were used.

### Preparation of Humanized TgVV, TgMM, and TgV180I Mice

The humanized TgVV mice expressing human wild-type PrP with VV at residue 129 (also termed TgWV) were prepared as described previously [17]; the TgMM mice (also called Tg40h) expressing human wild-type PrP with MM at residue 129 were generated by self-breeding of the previously reported Tg40 mice [18, 19]. For preparation of TgV180I mice, the transgene construct was generated by introducing the V180I mutation via PCR-based mutagenesis into the pHGHuPrP-129M plasmid, which was microinjected into fertilized FVB/NJ eggs, and planted into the oviducts of pseudopregnant CD-1 mice at the transgenic mouse facility of Case Western Reserve University (Cleveland, OH). Founder pups were screened by PCR of tail DNA. All founder mice carrying the transgenes were bred with FVB/Prnp^0/0^ mice to obtain Tg mice in PrP-null background. Transgenic PrP expression in the brain and other tissues of the Tg mice was examined by Western blot analysis using monoclonal antibody 3F4. All animal experiments in this study were approved by the Institutional Animal Use and Care Committee and the Institutional Biosafety Committee.

### Preparation of Recombinant Bank Vole PrP109M or PrP109I

The cloning of the bank vole PrP109M or PrP109I (BVPrP109M or BVPrP109I) genes were carried out based on the previously described [20]. The DNA coding for full-length BVPrP-109M and BVPrP-109I was amplified by PCR using a template plasmid of BVPrP-109M/pOPINE or BVPrP-109I/pOPINE. The amplification was carried out using oligonucleotides 5′ CGCGGATCCATGAAGAAGCGGCCAAAGCCTGG 3′ and 5′ CCCAAGCTTTTAGGAACTTCTCCCTTCGT 3′. The PCR product was digested with BamHI and HindIII and inserted into pET-28a (Novagen). The bank vole PrP109M or PrP109I recombinant protein expression and purification was done according to our previous study [21]. The *Escherichia coli* Rossetta (DE3) pLysS were transformed with full-length BVPrP-109M or BVPrP-109I for a large-scale production. The bacteria were induced at A600 = 0.6 by adding 1 mM isopropyl-b-d-thiogalactopyranoside (IPTG) and then subsequently grown at 37 °C for 5–6 h. Cells were collected by centrifugation (15 min at 15,000*g*). The bacterial pellets were re-suspended as 0.1 g of cell paste/milliliter in lysing buffer (10 mM Tris-HCl, 100 mM Na-PO4 buffer, pH 8.0). Mechanical disruption was used to lyse the cells and followed by centrifugation at 4 °C for 30 min at 15,000×*g*. The inclusion bodies were re-suspended in 6 M guanidine hydrochloride (GdnHCl), 10 mM Tris-HCl, 100 mM Na-PO4 buffer, and 10 mM β-mercaptoethanol (βME), pH 8.0, and sonicated on ice until completely solubilized. The recombinant bank vole PrP109M or PrP109I was refolded on the Ni-NTA column by running a GdnHCl gradient (from 6 M GdnHCl, 10 mM Tris-HCl, and 10 mM βME, pH 8.0, to 10 mM Tris-HCl and 100 mM Na-PO4 buffer, pH 8.0) at 1 mL/min. After wash, recombinant PrP was eluted with 10 mM Tris-HCl, 100 mM Na-PO4 buffer, and 500 mM imidazole, pH 5.8. The eluted soluble BVPrP fractions were loaded on a SDS/PAGE to evaluate protein purity and then pooled. The fractions only containing BVPrP were collected and dialyzed against 10 mM sodium phosphate buffer pH 5.8 and concentrated to 0.7 mg/mL. Protein aliquots were stored at − 80 °C until use.

### Preparation of Human or Mouse Brain Samples for Western Blotting, sPMCA, and RT-QuIC Assays

Frozen brain tissues from patients with VPSPr, fCJD^V180I^, fCJD^T183A^, sCJDMM1, and sCJDVV2, or normal subjects with PrP129-MM or PrP129-VV and from humanized transgenic mice of TgVV, TgMM, or TgV180I perfused, were collected and kept at − 80 °C. The samples were carefully cleaned with PBS for three times before the processing of each specimen in order to avoid blood contamination. For Western blotting, or sPMCA assay, Beads Beater was used to prepare tissue homogenates and the samples were then centrifuged at 500*g* for 5 min. The supernatant (S1) was transferred to a clean tube for future use while the pellet (P1) was discarded. For RT-QuIC analysis, the S1 fraction was diluted at 1:1 with 2 × conversion buffer containing 300 mM NaCl, 2% Triton X-100, and a complete protease inhibitor in PBS without Ca^2+^ and Mg^2+^ to prepare a 5% brain homogenate and then make serial dilution with 1 × N2 in 0.05%SDS/PBS as described [22].

### Serial PMCA Procedures

The preparation of PrP seeds and substrates as well as sPMCA was conducted as previously described [17, 23]. In brief, human or Tg mouse brain tissues were carefully dissected to avoid cerebellum and blood contamination as much as possible. Brain homogenate substrates from normal frozen brains were homogenized (10% *w*/*v*) in PMCA conversion buffer containing 150 mM NaCl, 1% Triton X-100, and 8 mM EDTA pH 7.4 and the complete protease inhibitor mixture cocktail (Roche) in PBS. The seeds of the brain tissue homogenates were prepared as the brain substrates described above. Tissue homogenates were centrifuged at 500*g* for 10 min at 4 °C and the supernatant (S1) fraction was collected as the substrate or centrifuged at 500*g* for 3 min for the seeds of brain samples. The substrates and seeds were kept at − 80 °C until use. Each seed was diluted in the substrate at the ratios from 1:12.5 to 1:100 (1 μL or 8 μL seed + 99 μL or 92 μL substrate) into 200-μL PCR tubes with 1 PTFE beads (diameter 3/32″) (Teflon, APT, RI). Twenty microliters of each mixture was taken and kept at − 20 °C as a non-PMCA control. The remaining mixture was subjected to serial PMCA (sPMCA). Each cycle comprised a 20-s elapse time of sonication at amplitude 85 (250 W; Misonix S3000 sonicator) followed by an incubation period of 29 min 40 s at 37 °C and each round of sPMCA consisted of 96 cycles. For the serial PMCA, 15 μL sample was taken from the last cycle and placed into 85-μL fresh normal brain substrates for a new round of amplification.

### RT-QuIC Analysis

RT-QuIC assay was conducted as previously described [20, 22, 24]. Briefly, the reaction mix was composed of 10 mM phosphate buffer (pH 7.4), 300 mM NaCl, 0.1 mg/mL recombinant bank vole PrP23-231, 10 μM thioflavin T (ThT), 1 mM ethylenediaminetetraacetic acid tetrasodium salt hydrate (EDTA), and 0.001% SDS. Aliquots of the reaction mix (98 μL) were loaded into each well of a 96-well plate (Nunc) and seeded with 2 μL of brain homogenate spinning at 2000*g* for 2 min at 4 °C as previously described [22]. The plate was then sealed with a plate-sealer film (Nalgene Nunc International) and incubated at 42 °C in a BMG FLUOstar Omega plate reader with cycles of 1-min shaking (700 rpm double orbital) and 1-min rest throughout the indicated incubation time. ThT fluorescence measurements (450 ± 10-nm excitation and 480 ± 10-nm emission; bottom read) were taken every 45 min. Four replicate reactions were seeded with the same dilution of an individual sample. The average fluorescence values per sample were calculated using fluorescence values from all four replicate wells regardless of whether these values crossed the threshold described below. At least 2 of 4 replicate wells must cross this threshold for a sample to be considered positive.

### Western Blotting

sPMCA-treated brain samples were subjected to treatment with PK at 100 μg/mL for 70 min at 45 °C with agitation prior to Western blotting. Samples were resolved either on 15% Tris-HCl Criterion pre-cast gels (Bio-Rad) for SDS-PAGE as described previously [25]. The proteins on the gels were transferred to Immobilon-P membrane polyvinylidene fluoride (PVDF, Millipore) for 2 h at 350 mA. For probing of PrP, the membranes were incubated for 2 h at room temperature with anti-PrP antibodies 3F4 at 1:40,000, 1E4 at 1:500, T2 at 1:8000, Bar209 at 1:6000, and V14 at 1:6000 dilution, as the primary antibody. Following incubation with horseradish peroxidase-conjugated sheep anti-mouse IgG at 1:4000 or donkey anti-rabbit IgG (for T2 only) at 1:6000 dilution, the PrP bands were visualized on Kodak film by ECL Plus as described by the manufacturer. PrP protein bands were measured by densitometric analysis and quantified using a UN-SCAN-IT Graph Digitizer software (Silk Scientific, Inc., Orem, Utah).

### Statistical Analysis

The statistical differences in intensity of PrP^Sc^ amplified by sPMCA among different groups detected by Western blotting were statistically analyzed using Student’s *T* test or ANOVA test to obtain *p* values for comparisons between two groups or multiple groups.

## Results

### PrP^Sc^ from VPSPr and fCJD^V180I^ to be examined for prion seeding activity lacks diglycosylated PrP^Sc^

**Fig. 1:**
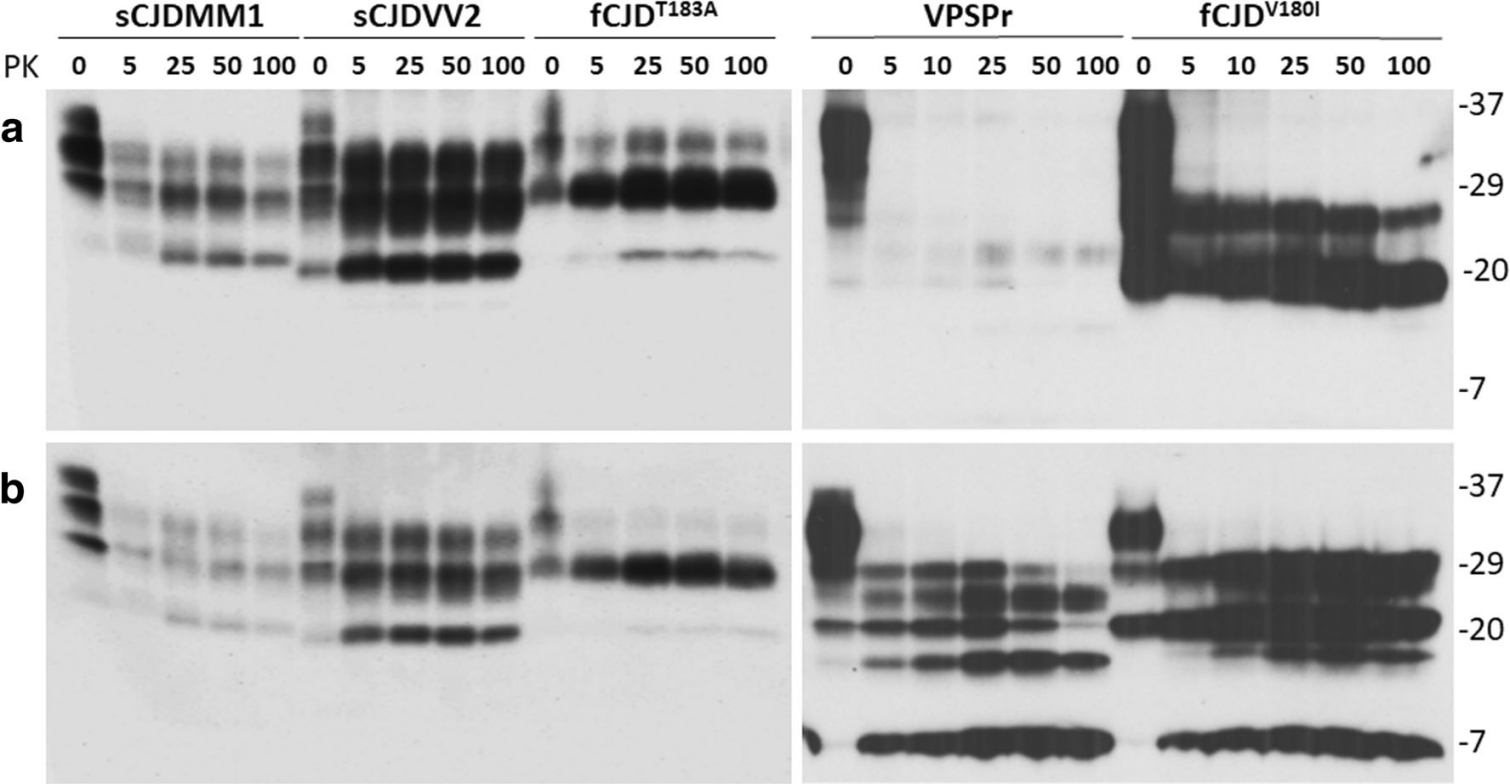
Comparison of PrP^Sc^ from VPSPr, fCJD^V180I^, fCJD^T183A^, sCJDMM1, and sCJDVV2. Representative Western blotting of untreated and treated PrP^Sc^ with different amounts of PK from sCJDMM1, sCJDVV2, fCJD^T183A^, VPSPrMV, and fCJD^V180I^ probed with 3F4 (a) and 1E4 (b). PrP^Sc^ from fCJD^V180I^ and VPSPr lacks the PK-resistant diglycosylated PrP^Sc^ on the Western blotting probed with 3F4 while it has diglycosylated PrP species prior to PK treatment. It contains monoglycosylated PrP migrating at ~ 26 kDa and un-glycosylated PrP migrating at ~ 20 kDa. On the 1E4 blot (b), additional small fragments migrating at ~ 23 kDa, ~ 17 kDa, and ~ 7 kDa were detected in the PK-treated samples from fCJD^V180I^ and VPSPr. Molecular weight markers are shown in kDa on the right side of the blots

We first wanted to confirm our previous finding in the samples to be examined in this study that PrP^Sc^ from the brain of patients with VPSPr and fCJD^V180I^ lacks the PK-resistant diglycosylated glycoform, detected by the 3F4 and 1E4 anti-PrP antibodies [4–6, 9]. On the 3F4 blot, in contrast to sCJD, the brain homogenates from VPSPr and fCJD^V180I^ patients exhibited only mono- and un-glycosylated PK-resistant PrP bands migrating at approximately 26 kDa and 20 kDa upon the treatment of brain homogenates with different amounts of PK ranging from 5 through 100 μg/mL (Fig. 1a). However, in the samples without PK treatment (0 μg/mL), the diglycosylated PrP was readily detectable not only in sCJD but also in fCJD^V180I^ and VPSPr (Fig. 1a). Our previous studies demonstrated that the lack of the PK-resistant diglycosylated PrP^Sc^ results from the missing PrP species diglycosylated and monoglycosylated at residue 181 that are not converted into the PK-resistant PrP^Sc^ molecule [4, 6]. The PK-resistant PrP^Sc^ from fCJD^T183A^ exhibited a predominant monoglycosylated PrP band as well as a barely detectable diglycosylated and an un-glycosylated PrP bands (Fig. 1a).

The epitopes of the 1E4 and 3F4 antibodies are next to each other on the protein, the former being composed of human PrP97-105 and the latter covering human PrP106-112 [13, 14], as shown in the schematic diagram of PrP structure and modifications in Fig. S1 [26–28]. We previously observed that the two antibodies have different immunoreactivity with distinct PrP^Sc^ molecules. For instance, compared to 3F4, 1E4 has a higher affinity for the sCJD PrP^Sc^ type 2 and a lower affinity for sCJD PrP^Sc^ type 1, in addition to its higher affinity for the unique ladder-like PrP^Sc^ in VPSPr and fCJD^V180I^ [3–6]. According to the previous sequencing study, the PK-resistant PrP^Sc^ type 2 fragment starts at residue 97 [29], the first amino acid of the 1E4 epitope. Thus, it is most likely that the higher immunoreactivity of the 1E4 antibody than that of the 3F4 antibody is directly attributable to the well-exposed 1E4 epitope on the PrP^Sc^ type 2 fragment.

To confirm the particular gel profiles of PrP^Sc^ from VPSPr and fCJD^V180I^, we next probed the blots with the 1E4 antibody. Indeed, although the equal amounts of brain homogenates were loaded, compared to 3F4, 1E4 showed a greater intensity for sCJD PrP^Sc^ type 2 while a weaker intensity for sCJD PrP^Sc^ type 1 (Fig. 1b, left two panels). Moreover, in addition to the two PK-resistant PrP^Sc^ bands migrating at ~ 26 and ~ 20 kDa, 1E4 detected three additional PK-resistant PrP fragments migrating at ~ 23, ~ 17, and ~7 kDa in the samples from fCJD^V180I^ and VPSPr, exhibiting the unique ladder-like PK-resistant PrP^Sc^ bands (Fig. 1b). They were not detectable in the samples from sCJDMM1 and sCJDVV2 and also not in the samples from fCJD^T183A^ (Fig. 1b). Since the PrP^T183A^ mutation has been shown to completely abolish the *N*-linked glycosylation at residue 181 [7–9], the faint diglycosylated PrP^Sc^ detected by both 3F4 and 1E4 is expected to be from the wild-type allele (Fig. 1a, b) [7, 9].

### PrP^Sc^ from VPSPr and fCJD^V180I^ Preferentially Seeds Human Brain–Derived PrP^C^-129MM Substrate by sPMCA, Forming Diglycoform-Containing PrP^Sc^

Given the unique PrP^Sc^ profile and low transmissibility of VPSPr and fCJD^V180I^, it would be important to determine whether the PrP^Sc^ molecules from these diseases can convert normal PrP^C^ from healthy human brain homogenates and what types of PrP^Sc^ can be generated in vitro compared to PrP^Sc^ from the most common sCJD. We conducted serial protein misfolding cyclic amplification (sPMCA) of PrP^Sc^ from brain homogenates of VPSPr-129MM (VPSPrMM), VPSPr-129VV (VPSPrVV), VPSPr-129MV (VPSPrMV), and fCJD^V180I^-129MM using two types of PrP^C^ from healthy human brain homogenate with either 129MM or 129VV polymorphism as the substrates. sPMCA is a highly efficient in vitro amplification assay that has been shown to be able to faithfully mimic prion conversion by continuously seeding PrP^Sc^ in normal PrP^C^ substrate [23]. PrP^Sc^ from sCJDMM1, sCJDVV2, and fCJD^T83A^ was used as controls. It is known that the PrP polymorphism at residue 129 of the protein can significantly affect the efficiency of PrP^C^-PrP^Sc^ conversion. Thus, the PrP^Sc^ seeds from VPSPr carrying different 129-polymorphisms and the PrP^C^ substrates from healthy human brain homogenates with either 129MM (hMM) or 129VV (hVV) were examined by sPMCA.

**Fig. 2:**
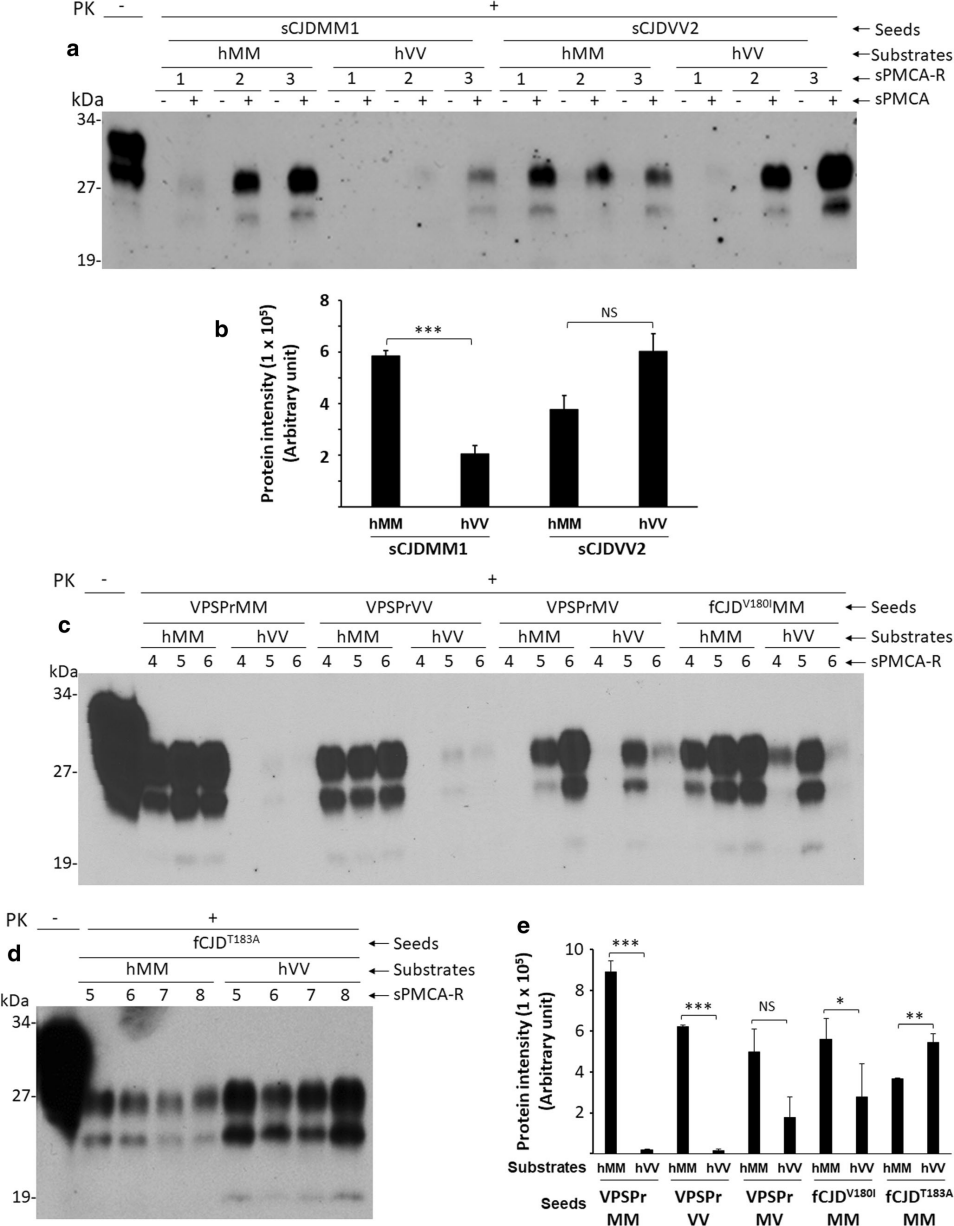
Serial PMCA of PrP^Sc^ from sCJD, VPSPr, fCJD^V180I^, and fCJD^T183A^ in human brain homogenate substrates. Representative Western blotting of PrP^Sc^ from sCJDMM1 and sCJDVV2 (**a**), VPSPr, fCJD^V180I^, and fCJD^T183A^ (**c**, **d**) amplified with 1–3, 1–6, or 1–8 rounds of sPMCA in human brain homogenate substrates from non-CJD MM (hMM) or VV (hVV) probed with the 3F4 antibody. sPMCA-R, sPMCA rounds. Molecular weight markers are shown in kDa on the left side of the blots. PK, proteinase K. **b**, **e** Bar graph showing the quantitative analyses of the intensity of PrP^Sc^ amplified by sPMCA after densitometric scanning. *p < 0.05; **p < 0.01; ***p < 0.001; NS, not statistically significant

Amplification of PrP^Sc^ from sCJDMM1 or sCJDVV2 was observed in all three rounds of sPMCA-treated samples but not in non-sPMCA-treated control samples conducted with the seed-substrate polymorphism-matched and unmatched sPMCA (Fig. 2a, b). The amplification efficiency of sCJDMM1 PrP^Sc^ was significantly greater in seed-substrate-matched than unmatched sPMCA (MM-MM vs MM-VV, *p* < 0.001) while there was no significant difference in the amplification efficiency of sCJDVV2 between the seed-substrate-matched and unmatched sPMCA (VV-VV vs VV-MM, *p* > 0.05) (Fig. 2a, b, Table S1).

After 6 rounds of sPMCA, PrP^Sc^ from three genotypes of VPSPr including 129-MM, 129-VV, and 129-MV was all amplified in normal human brain homogenates hMM (Fig. 2c, e). In contrast, in hVV, PrP^Sc^ from VPSPrMM or VPSPrVV exhibited significantly low efficiency while the PrP^Sc^ amplification from VPSPrMV or fCJD^V180I^ varied (Fig. 2c, e). The amplification of PrP^Sc^ from VPSPrMM and VPSPrVV was significantly higher in hMM than in hVV substrate (*p* < 0.001), whereas no significant difference was observed for VPSPrMV (*p* > 0.05) (Fig. 2c, e). PrP^Sc^ from fCJD^V180I^ was amplified in both hMM and hVV substrates while it showed significantly stronger amplification in hMM than in hVV (Fig. 2c, e). Notably, PrP^Sc^ from fCJD^T183A^ was amplified efficiently with 5–8 rounds of sPMCA in both hMM and hVV; the amplification efficiency was significantly higher in hVV than in hMM (*p* < 0.01) (Fig. 2d, e), which was different from VPSPr, fCJD^V180I^, or sCJD. ANOVA analysis showed no differences in PrP^Sc^ amplification between different seeds but significant differences between substrates (Table S1).

In sum, like sCJDMM1, PrP^Sc^ from three genotypes of VPSPr and fCJD^V180I^ was all amplified in the hMM substrate while no or less amplification in the hVV substrate. PrP^Sc^ from sCJDVV2 or fCJD^T183A^ showed increased amplification in hVV than in hMM. In contrast to sCJD whose PrP^Sc^ amplification could be observed at the first round of sPMCA, PrP^Sc^ was not amplified until 4 or 5 rounds of sPMCA in VPSPr and fCJD, suggesting that the prion seeding activity in glycoform-deficient PrP^Sc^ from VPSPr and the two fCJD cases was lower than that from sCJD. Most surprisingly, all amplified PK-resistant PrP^Sc^ showed a predominant diglycosylated PrP isoform, although the PrP^Sc^ seeds display no, or significantly decreased, such isoform in VPSPr, fCJD^V180I^, and fCJD^T183A^.

### PrP^Sc^ of VPSPr and fCJD^V180I^ Favorably Seeds Tg Mice–Derived Human PrP^C^-129VV Substrate by sPMCA, Also Forming Diglycoform-Containing PrP^Sc^

Human PrP^C^ from the brain homogenate of humanized transgenic (Tg) mice expressing human PrP has been widely used as a substrate for amplification of human PrP^Sc^ by sPMCA in vitro [17, 30]. To determine whether the glycoform-deficient PrP^Sc^ from VPSPr and fCJD^V180I^ is amplifiable in different humanized Tg mouse brain homogenates, their PrP^Sc^ molecules were subjected to sPMCA in four different Tg mouse brain homogenates, respectively, expressing human PrP-129VV (TgVV), PrP-129MM (TgMM), PrP^V180I^ (Tg180), or in vitro mixed brain homogenate from TgMM and Tg180 mice (Tg180/TgMM). Since the PrP^V180I^ mutation is characterized with the deposition in the brain of fCJD^V180I^ of the unique PK-resistant PrP^Sc^ that has the gel profile similar to that of PrP^Sc^ from VPSPr, we generated humanized Tg mice expressing human PrP^V180I^ to determine how the mutation affects the PrP^Sc^ formation in vitro.

**Fig. 3:**
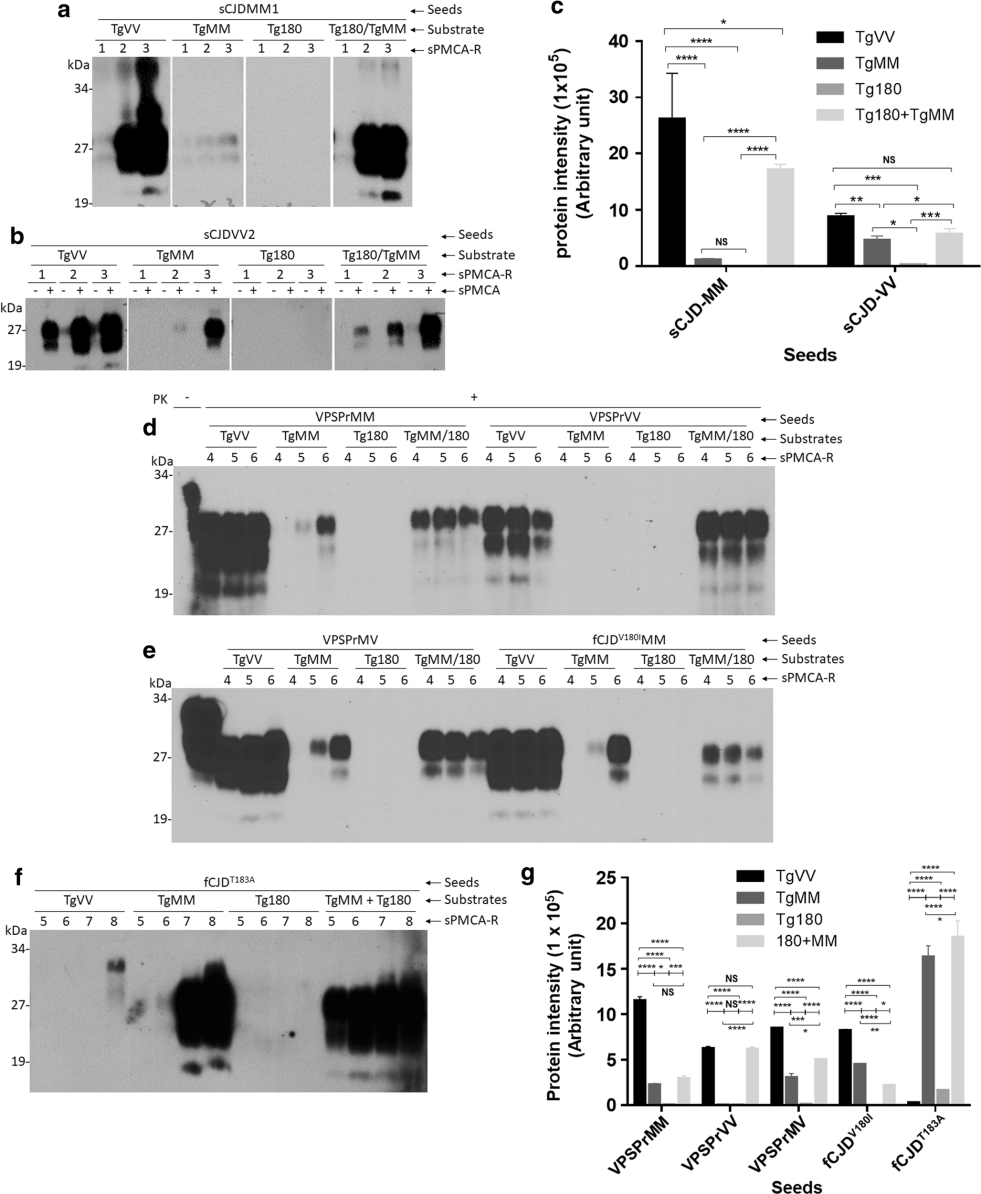
Serial PMCA of PrP^Sc^ from sCJD, VPSPr, fCJD^V180I^, and fCJD^T183A^ in transgenic mouse brain homogenates. Representative Western blotting of PrP^Sc^ from sCJDMM1 (**a**); sCJDVV2 (**b**); and VPSPr, fCJD^V180I^, and fCJD^T183A^ (**d**, **e**, **f**) amplified with 1–3, 1–6, or 1–8 rounds of sPMCA in humanized transgenic mouse brain homogenates from TgVV, TgMM, Tg180, or TgMM + Tg180 mouse line prior to PK digestion and Western blotting probed with the 3F4 antibody. sPMCA-R, sPMCA rounds. PK, proteinase K. **c**, **g** Bar graph showing the quantitative analyses of the intensity of PrP^Sc^ amplified by sPMCA after densitometric scanning. *p < 0.05; **p < 0.01; ***p < 0.001; ****p < 0.0001; NS, not statistically significant

We first determined amplification of classic PrP^Sc^ from sCJDMM1 or sCJDVV2 using sPMCA with the Tg mouse brain homogenate substrates. PrP^Sc^ from both sCJDMM1 and sCJDVV2 was amplified in the TgVV and TgMM substrates, respectively. The efficiency of amplification was significantly higher in TgVV than in TgMM substrate from both sCJDMM1 and sCJDVV2 PrP^Sc^ seeds (*p* < 0.01 or *p* < 0.0001) (Fig. 3a, b, c, Table S1). Interestingly, although PrP^Sc^ of sCJDMM1 or sCJDVV2 was virtually not amplified in the Tg180 substrate and less amplifiable in the TgMM substrate along, it was highly efficiently amplified in the in vitro mixed substrate of TgMM and Tg180 brain homogenate (*p* < 0.0005 or *p* < 0.0001) (Fig. 3a, b, c, Table S1).

Similar to PrP^Sc^ from sCJD, the amplification of PrP^Sc^ of all three genotypes of VPSPr and fCJD^V180I^ was highly efficient in the TgVV than in the TgMM substrate (*p* < 0.0001) (Fig. 3d, e, g, Table S1). Also no amplification was found using the brain homogenate as the substrate from Tg180 mice, although the level of PrP^C^ in the brain of Tg180 was similar to that of PrP^C^ in the brain of TgVV and TgMM (Fig. S2). Interestingly, amplification was rescued when TgMM substrate was mixed with the Tg180 mouse brain homogenate substrate (Fig. 3d, e). We also noticed that although the gel profiles of PrP^Sc^ seeds are different among different genotypes of VPSPr, sCJDMM1, and sCJDVV2, a similar pattern of PrP^Sc^ was generated by sPMCA using PrP^C^ from the TgVV brain homogenate as the same substrate, (Fig. 3a, b, d, e, Table S1), suggesting that the PrP^C^ substrate may determine the gel profile of newly generated PrP^Sc^ by sPMCA.

After 5–8 rounds of sPMCA, amplification efficiency of PrP^Sc^ from fCJD^T183A^ was significantly higher in TgMM than in TgVV substrate (*p* < 0.0001) (Fig. 3f, g), which was opposite to the amplification efficiency of PrP^Sc^ from VPSPr and fCJD^V180I^ (Fig. 3d, e, g). Notably, the similar opposite effect of PrP^T183A^ on the PrP^Sc^ amplification in the two Tg mouse–derived substrates between fCJD^T183A^ and all other prion diseases examined above including sCJD, VPSPr, and fCJD^V180I^ was also observed in human brain–derived PrP^C^ substrates (Fig. 2). Again, no PrP^Sc^ of fCJD^T183A^ was amplified in the Tg180 substrate but amplification was rescued when TgMM mouse brain homogenate was mixed with Tg180 brain homogenate (Fig. 3f). Although PrP^Sc^ from VPSPr, fCJD^V180I^, and fCJD^T183A^ contains virtually no diglycosylated PrP^Sc^, the same as in human brain–derived PrP^C^ substrate, a dominant diglycosylated PrP^Sc^ was amplified in the Tg mouse brain–derived PrP^C^ substrates as did PrP^Sc^ from sCJD (Fig. 3a, b, d, e, f, Table S1).

Taken together, as in the human brain–derived PrP^C^ substrate, PrP^Sc^ from VPSPr and fCJD^V180I^ was also amplified in the Tg mouse–derived human PrP^C^ substrate and the amplified PrP^Sc^ contained a dominant diglycosylated PrP species. However, unlike the human brain PrP^C^ substrate, the TgVV substrate was more susceptible to be recruited into PrP^Sc^ than the TgMM by sCJD, VPSPr, and fCJD^V180I^ by sPMCA. Like in the human brain substrate, PrP^Sc^ from fCJD^T183A^ showed the effect of the 129-polymorphsim on PrP^Sc^ amplification opposite to that from sCJD, VPSPr, and fCJD^V180I^. Remarkably, while PrP^Sc^ from none of the four human prion diseases was able to seed the Tg180 substrate alone, it was amplified only in the substrate of combination of TgMM and Tg180 substrates.

### No Small PK-Resistant PrP^Sc^ Fragments Are Amplified by sPMCA from VPSPr and fCJD^V180I^ in Both Human- and Humanized Tg Mouse–Derived PrP^C^ Substrates

In addition to the lack of diglycosylated PrP and the PrP molecule monoglycosylated at residue 181 shown by the 3F4 antibody and other anti-PrP antibodies including the 1E4 antibody, another unique feature of the gel profiles of PrP^Sc^ from VPSPr and fCJD^V180I^ is the formation of the ladder-like five PK-resistant PrP^Sc^ bands with extra small fragments detected by the 1E4 antibody [4–6, 31], as shown in Fig. 1. Moreover, using glycoform-specific anti-PrP antibodies Bar209 and V14, we identified the PrP molecules with *N*-linked glycosylation specifically at residue 181 that are not converted into PK-resistant PrP^Sc^ [4]. To determine whether the PrP^Sc^ amplified by sPMCA in TgVV, TgMM, Tg180, or the mixture of TgMM and Tg180 substrate contains those particular small PK-resistant fragments migrating at ~ 23 kDa, ~ 17 kDa, and ~ 7 kDa, we probed the amplified PrP^Sc^ with different anti-PrP antibodies.

**Fig. 4:**
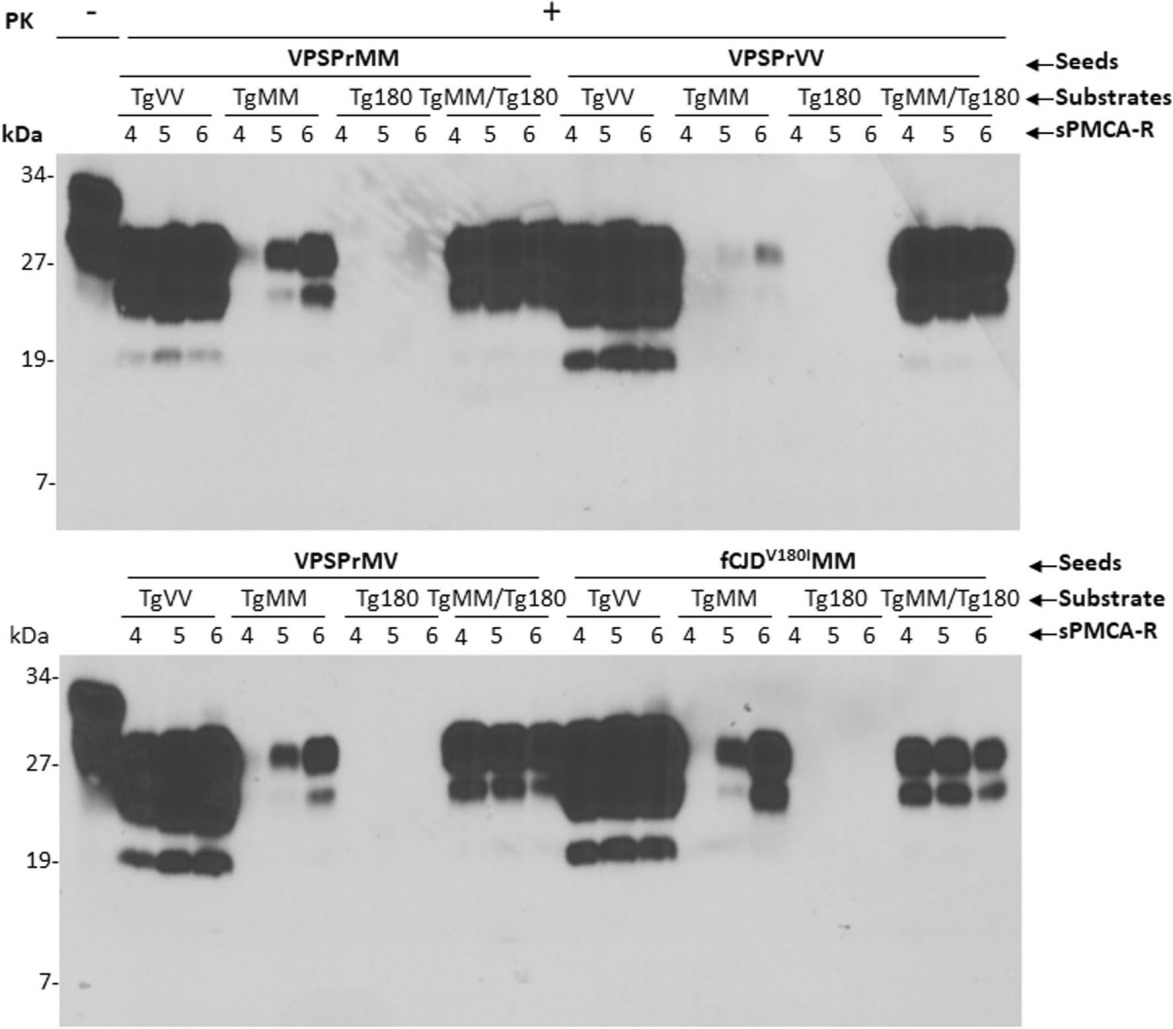
Serial PMCA of PrP^Sc^ from VPSPr and fCJD^V180I^ in transgenic mouse brain homogenates. Representative Western blotting of PrP^Sc^ amplified with 4–6 rounds of sPMCA by seeding PrP^Sc^ of VPSPrMM, VPSPrVV, VPSPrMV, or fCJD^V180I^ in humanized transgenic mouse brain homogenates from TgVV, TgMM, Tg180, or TgMM + Tg180 mouse line (b and c). The blots were probed with the 1E4 antibody. sPMCA-R, sPMCA rounds. Molecular weight markers are shown in kDa on the left side of the blots. PK, proteinase K

When the PrP^Sc^ molecule amplified with the VPSPr and fCJD^V180I^ seeds in the hMM or hVV substrate was probed with the 1E4 antibody, the PrP gel profiles observed were virtually the same as those detected by the 3F4 antibody without extra small PK-resistant PrP^Sc^ fragments (Fig. 4a). The observable differences between the two blots included that the intensity of the un-glycosylated PrP^Sc^ band from all VPSPr and fCJD^V180I^ amplified in hMM or hVV was increased on the 1E4 blot compared to the 3F4 blot and the amplified PrP^Sc^ in the hVV substrate became readily detectable with 1E4 (Fig. 4a with 1E4 vs Fig. 2c with 3F4). When PrP^Sc^ was amplified in the TgVV or TgMM mouse substrate, notably, no typical three small PK-resistant PrP^Sc^ were detected (Fig. 4b). Since it is known that 1E4 has a higher affinity for PrP^Sc^ type 2 than PrP^Sc^ type 1 compared to 3F4 [14], we expected that more PrP^Sc^ type 2 was detected by 1E4 from PrP^Sc^ amplified in the TgVV substrate compared to 3F4. To confirm this, we used another antibody named Tohoku 2 (T2) that specifically detects PrP^Sc^ type 2 to probe the blot [15]. As shown in Fig. S3, similar to 1E4, the intensity of the PK-resistant un-glycosylated PrP was increased compared to that shown by the 3F4 antibody, although there were some smear bands migrating under ~ 19 kDa in the TgVV or TgMM + Tg180 seeded with PrP^Sc^ of VPSPrMM or VPSPrVV.

### PrP^Sc^ of VPSPr and fCJD^V180I^ Amplified in Human Brain– or Humanized Tg Mouse–Derived PrP^C^ Substrates Contains Intact Mono- and Diglycosylated PrP Species

To determine whether there is any glycoform deficiency of PrP^Sc^ amplified by sPMCA, we detected the sPMCA-amplified PrP^Sc^ with glycoform-specific anti-PrP antibodies Bar 209 and V14 [4, 16]. The Bar209 antibody specifically recognizes the PrP molecule monoglycosylated at residue 197 while the V14 antibody specially recognizes the PrP molecule monoglycosylated at residue 181; both of them detect the un-glycosylated PrP molecule [4, 16]. When the PrP^Sc^ amplified in different brain homogenate substrates was probed with the two antibodies, the two glycoforms were detected by the two antibodies (Fig. S4), suggesting that unlike the PrP^Sc^ seeds themselves from VPSPr and fCJD^V180I^, both glycoforms were converted into PrP^Sc^ from various PrP^C^ substrates by sPMCA.

### In Vitro Prion Seeding Activity of Glycoform-Deficient PrP^Sc^ from VPSPr and fCJD^V180I^ Is Detectable by RT-QuIC Assay

**Fig. 5:**
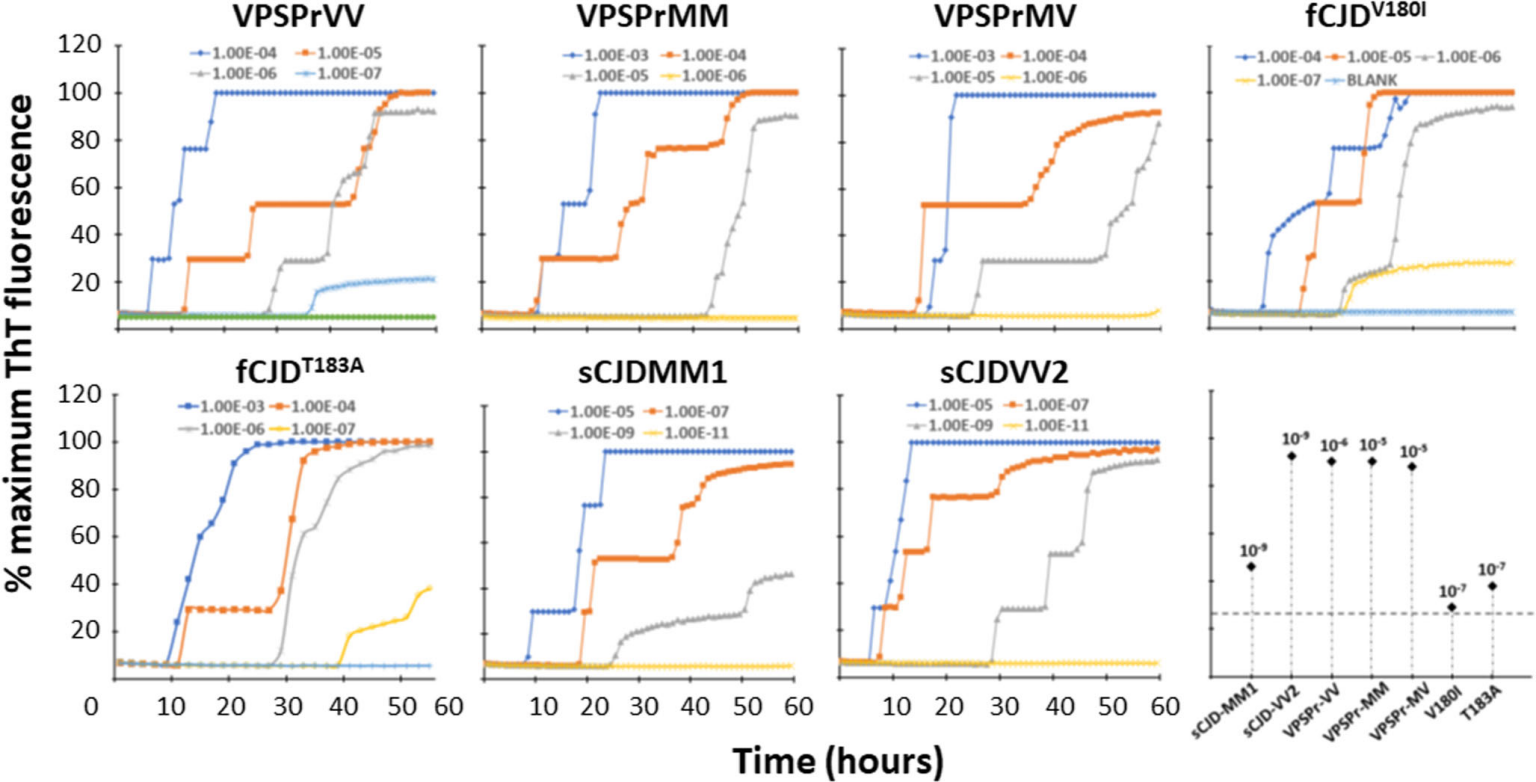
RT-QuIC analysis of PrP^Sc^ from VPSPr, fCJD^V180I^, fCJD^T183A^, sCJDMM1, and sCJDVV2 with the recombinant bank vole PrP109I. PrP^Sc^ from VPSPr, fCJD^V180I^, fCJD^T183A^, sCJDMM1, and sCJDVV2 was seeded in recombinant bank vole PrP23-231 prior to RT-QuIC assay. The prion seeding activity was measured until 60 h. Different dilution of brain homogenates from infected human brains was tested from 10^−3^ to 10^−11^ according to their seeding activity. The panel at the lower right corner of the figure is the scatter graph for the comparison of the maximal dilution to be detected by RT-QuIC

RT-QuIC is another in vitro ultrasensitive assay to amplify and quantify PrP^Sc^ by measuring its seeding activity [32]. Prion-seeding activity is expected to reflect the ability of PrP^Sc^ to replicate in the presence of the PrP^C^ substrate, characteristic of infectious prions [22, 24, 32]. Unlike sPMCA, RT-QuIC assay uses the recombinant PrP as the substrate instead of non-infected brain homogenate and monitors the aggregation-triggered increase in thioflavin T fluorescence in real time [24, 32]. Our RT-QuIC end-point titration assay revealed that the seeding activity of PrP^Sc^ from VPSPr and fCJD^V180I^ in the recombinant bank vole PrP23-231 substrate was approximately 10^2^- to 10^5^-fold lower than that of PrP^Sc^ from sCJDMM1 and sCJDVV2 (Fig. 5). Among the three genotypes of VPSPr, fCJD^V180I^, and fCJD^T183A^, the prion seeding activity was highest in fCJD^T183A^, followed by VPSPrVV, fCJD^V180I^, VPSPrMM, and VPSPrMV according to the seeding activity at the highest dilution (Fig. 5). The latter two (VPSPrMM and VPSPrMV) exhibited the similar prion seeding activity.

## Discussion

The key molecular event in the pathogenesis of various animal and human prion diseases is the conversion of PrP^C^ into PrP^Sc^. The molecular mechanism underlying the structural conversion of the two isoforms remains poorly understood, which prevents not only our understanding of pathogenesis and transmission of prion diseases between different species but also the development of efficient therapeutics for prion diseases. For instance, the species barriers are widely present in transmission of prion diseases among different species and are believed to result from a complex interplay of primary amino acid sequence, glycoform patterns, and three-dimensional structure of the PrP molecule [3].

Several lines of evidence have suggested that the individual PrP glycoforms may affect the efficiency of PrP^C^-to-PrP^Sc^ conversion even within the same species and individuals. Our previous work has shown, for the first time, that similar to fCJD^T183A^, in which the PrP^T183A^ mutation eliminates the *N*-linked glycosylation at residue 181, the PrP molecules with *N*-linked glycosylation at residue 181, including both di- and monoglycosylated species, are not converted into PK-resistant PrP^Sc^ in sporadic VPSPr and familial CJD associated with PrP^V180I^ mutation [4, 6]. However, our study also revealed that in contrast to fCJD^T183A^, both VPSPr and fCJD^V180I^ exhibit three intact PrP glycoforms prior to PK digestion [4, 9]. Indeed, the glycoform profiles of PrP^V180I^ mutation from transfected M17 cell lines and humanized Tg mice expressing human PrP with V180I mutation also showed no difference from those of wild-type PrP expressed in similar cell lines and humanized Tg mice [4]. Notably, we observed that PK-resistant PrP^V180I^ from the cultured M17 cells contained diglycosylated PrP species, which made us think that the deficiency in conversion of PrP^C^ into PrP^Sc^ may not be directly attributable to the PrP^V180I^ mutation alone and there might be other factors that are involved in mediating the formation of this unique PrP^Sc^ isoform in vivo. Our previous findings raised two possibilities: first, the glycoform-selective prion formation observed in the brain of patients with VPSPr or fCJD^V180I^ may involve dominant-negative inhibition caused by the interaction between misfolded and normal PrP molecules; second, one or more co-factors may be operating in VPSPr and fCJD^V180I^ and the co-factors may prevent conversion of the PrP^C^ molecules with *N*-linked glycosylation at residue 181 into PrP^Sc^ including both di- and monoglycosylated species [4, 6].

In addition, it is possible that the presence of the multiple PK-resistant PrP^Sc^ fragments in VPSPr and fCJD^V180I^ may be associated with the dysfunction of the endoproteolytic processing event in vivo. For instance, a small C-terminal PK-resistant PrP^Sc^ termed C3 migrating at ~ 7 kDa that was believed to be generated by the *γ*-cleavage was significantly increased in the brain of sCJD patients [33]. The ~ 7-kDa fragment, one of the three small PK-resistant PrP^Sc^ fragments detected in VPSPr and fCJD^V180I^, is detected by 1E4 with the epitope localized between residues 97 and 105. Thus, this fragment is expected to be different from the C3 fragment generated by the *γ*-cleavage but more like the 7-kDa PrP observed in GSS.

The serial PMCA is able to faithfully amplify PrP^Sc^ in vitro [23, 34]. Using this ultrasensitive method, we observed that PrP^Sc^ from VPSPr and fCJD^V180I^ was amplified very efficiently using the brain homogenate substrates from TgVV, non-CJD patients with PrP-129MM, and mixed brain homogenates with Tg180 and TgMM as well as less efficiently using TgMM and non-CJD patients with PrP-129VV. Currently, we do not know the exact reason why the PrP^C^ from TgVV substrate is more susceptible to sCJDMM1, VPSPr, and fCJD^V180I^MM than that from TgMM, except for fCJD^T183A^. It would be interesting to compare sPMCA assay with animal-based bioassay with these cases in the future. No PrP^Sc^ amplification was found when brain homogenate from Tg180 alone was used as the substrate. PrP^Sc^ from fCJD^T183A^ was also amplified using human hMM or hVV, TgMM, or TgMM + Tg180. Moreover, like sCJDMM1 or sCJDVV2, PrP^Sc^ amplified from VPSPr and fCJD^V180I^ surprisingly showed a dominant diglycosylated PrP^Sc^ isoform.

Our finding is consistent with the recent observation by Peden et al. [35] in which they observed the generation of a ~ 30-kDa band corresponding to PK-resistant diglycosylated PrP^Sc^ after PMCA of PrP^Sc^ from brain homogenates of VPSPr patients using human brain homogenate as the substrate. Notably, sPMCA of PrP^Sc^ from fCJD^T183A^ also generated a dominant diglycosylated PrP^Sc^ isoform. The PrP^T183A^ mutation has been shown to specifically eliminate the first *N*-linked glycosylation site of the protein so that the mutant PrP cannot have either the di- or the monoglycosylated form at residue 181. Bearing this in mind, it is conceivable that the PK-resistant diglycosylated PrP amplified from fCJD^T183A^ was most likely originated from the wild-type PrP^Sc^ since most of the fCJD patients, if not all of them, carry both mutant and wild-type alleles. Indeed, a small amount of PK-resistant diglycosylated PrP^Sc^ was observed in the brain samples from a patient with fCJD^T183A^, which was considered to derive from the wild-type allele [8]. Although it is unclear at the present where the diglycosylated PrP^Sc^ exactly comes from and why the diglycosylated PrP^Sc^ is highly efficiently generated in sPMCA reactions seeded by VPSPr and fCJD^V180I^ brain homogenates, it is likely that the amplified diglycosylated PrP^Sc^ could be converted from the normal wild-type PrP as does PrP^Sc^ in fCJD^T183A^. On the other hand, the extra small PK-resistant PrP fragments detected specifically by the 1E4 antibody were not amplified in either human or mouse brain homogenates, consistent with the observation as well by Peden et al. [35]. The finding of no seeding activity of these 1E4-detected PrP^Sc^ small fragments by sPMCA may echo the observation of the low, or lack of, transmissibility of VPSPr observed by bioassays [10, 11].

While PrP^Sc^ from VPSPr showed highly efficient amplification in the brain homogenate of TgVV, similar to that from fCJD^V180I^ and sCJD, it had a poor amplification in the brain homogenate of TgMM compared to that from the two fCJD and two sCJD cases. Although the amplification of PrP^Sc^ from all three genotypes of VPSPr with brain homogenates from TgMM or Tg180 mice alone was poor or even not at all, it was significantly increased by mixing the brain homogenates from the two Tg mice, especially for PrP^Sc^ from VPSPr-129VV. This result seemed to contradict the observation found in the brain of patients with VPSPr, where we expected that the interaction between wild-type and mutant PrP^V180I^ in the brain may prevent the conversion of the PrP^C^ molecules glycosylated at residue 181 to the PK-resistant PrP. Telling et al. [36] reported that the presence of wild-type PrP could delay or prevent prion formation initiated by the mutant PrP. Also, Noble et al. [37] observed that recombinant wild-type PrP in trans inhibited the spontaneous formation of a PK-resistant recombinant mutant PrP. Therefore, it is most likely that PrP^Sc^ from VPSPr and fCJD^V180I^ could represent a unique prion strain that behaves differently from other common human prion strains.

RT-QuIC assay is another ultrasensitive approach for conversion of normal PrP into PrP aggregates in vitro [22, 24, 32]. It has been found that RT-QuIC assay is able to differentiate prion strains [22, 24, 35]. Unlike sPMCA, it uses recombinant PrP molecules instead of normal brain homogenates as the substrates and involves shaking instead of sonication to trigger the protein conversion process. The other feature of RT-QuIC is that the sequence barriers of the PrP^Sc^-PrP^C^ seeding are not so strict, which does seem to be different from sPMCA. For instance, many prion strains from different species can have fairly efficient conversion by RT-QuIC with substrates of recombinant hamster or bank vole PrP molecule. On the other hand, the conversion efficiency of PrP by sPMCA is mostly determined by the similarity in protein sequences, which is consistent with in vivo bioassays. Using the RT-QuIC assay, prion seeding activity was found using all genotypes of VPSPr, fCJD^V180I^, and fCJD^T183A^. Our RT-QuIC end-point titration assay indicated that the prion seeding activity was approximately 10^2^- to 10^5^-fold lower in VPSP and fCJD^V180I^ than in sCJDMM1 and sCJDVV2, which is consistent with the observation by Peden et al. [35] in which they observed a lower prion seeding activity in VPSPr than in sCJD.

In conclusion, our current findings indicate that like sCJD, the glycoform-deficient PrP^Sc^ from VPSPr and fCJD^V180I^ can be amplified as the typical PrP^Sc^ with intact diglycosylated species using sPMCA. It also exhibited prion seeding activity despite the lower amount than that in sCJD. The amplification efficiency of the glycoform-deficient PrP^Sc^ in vitro was enhanced by the interaction of brain homogenates from wild-type and mutant PrP^C^ substrates. The extra three small PK-resistant PrP^Sc^ fragments were not be amplified by sPMCA in vitro, suggesting that those PrP^Sc^ species may not be highly infectious, which is consistent with the observations by in vivo animal-based bioassay reported previously. On the other hand, the inability of in vitro amplification to duplicate the unique PrP^Sc^ gel profile of VPSPr and fCJD^V180I^ with either human or humanized Tg mouse brain substrate suggests that the formation of glycoform-selective and the ladder-like PK-resistant PrP^Sc^ may be associated with an unidentified factor in the affected human brain. It would be important to exclude the possibility in the future that the unknown factor could be a second component that is able to cause prion disease, as shown in other neurodegenerative diseases such as Alzheimer’s disease and amyotrophic lateral sclerosis in which there are more than one gene that are involved in the pathogenesis of the diseases.

## Electronic Supplementary Material

ESM 1 (DOC 925 kb)
