# Supplementary material for: In Vitro Seeding Activity of Glycoform-Deficient Prions from Variably Protease-Sensitive Prionopathy and Familial CJD Associated with PrPV180I Mutation
Source: Mol Neurobiol. 2019 Jan 5;56(8):5456–69. doi: 10.1007/s12035-018-1459-0 (PMC6614145; doi:10.1007/s12035-018-1459-0)
Supplement: Supplementary file 1 — (DOC 925 kb) [file 12035_2018_1459_MOESM1_ESM.pdf]

# *In vitro* seeding activity of glycoform-deficient prions from variably protease-sensitive prionopathy and familial CJD associated with $PrP^{V180I}$ mutation

Zerui Wang[1,2], Jue Yuan[2], Pingping Shen[1], Romany Abskharon[3], Yue Lang[1,2], Johnny Dang[2], Alise Adornato[2], Ling Xu[2], Jiafeng Chen[1], Jiachun Feng[1], Mohammed Moudjou[4], Tetsuyuki Kitamoto[5], Jan Langeveld[6], Brian Appleby[2,7,8], Jiyan Ma[3], Qingzhong Kong[2,7,8], Robert B. Petersen[2,9*], Wen-Quan Zou[1,2,7,8,10*], Li Cui[1*]

[1]Department of Neurology, The First Hospital of Jilin University, Changchun, Jilin Province, the People's Republic of China.

[2]Department of Pathology, Case Western Reserve University School of Medicine, Cleveland, Ohio, USA.

[3]Center for Neurodegenerative Science, Van Andel Research Institute, Grand Rapids, 49503 Michigan, USA.

[4]Virologie Immunologie Moléculaires, INRA, Jouy-en-Josas, France.

[5]Center for Prion Diseases, Tohoku University Graduate School of Medicine, 2-1 Seiryo-machi, Aoba-ku, Sendai 980-8575, Japan.

[6]Wageningen BioVeterinary Research, Houtribweg 39, Lelystad, the Netherlands.

[7]National Prion Disease Pathology Surveillance Center, Case Western Reserve University School of Medicine, Cleveland, Ohio, USA.

[8]Department of Neurology, Case Western Reserve University School of Medicine, Cleveland, Ohio, USA.

[9]Foundation Sciences, Central Michigan University College of Medicine, Mount Pleasant, Michigan, USA.

[10]State Key Laboratory for Infectious Disease Prevention and Control, National Institute for Viral Disease Control and Prevention, China Center for Disease Control and Prevention, Beijing, China.

*Corresponding: WQZ at wxz6@case.edu; or LC at chuili1967@126.com; or RBP at robert.petersen@cmich.edu

**Table S1**

**Table S1 ANOVA analysis**

| Groups | Comparison | p values |
|---|---|---|
| Fig. 2b | Seeds | >0.05 |
| | Substrates | >0.05 |
| | Interaction | <0.0005 |
| Fig. 2e | Seeds | >0.05 |
| | Substrates | <0.0001 |
| | Interaction | <0.0001 |
| Fig. 3c | Seeds | <0.0005 |
| | Substrates | <0.0001 |
| | Interaction | <0.0001 |
| Fig. 3g | Seeds | <0.0001 |
| | Substrates | <0.0001 |
| | Interaction | <0.0001 |

**Fig. S1**

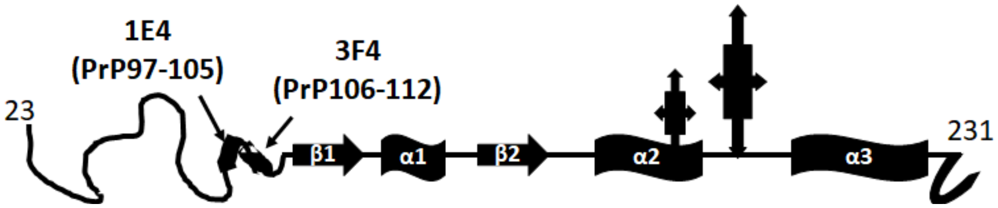


Fig. S1 Schematic diagram of locations of the 1E4 and 3F4 epitopes on PrP and structure of human PrP. The mature human PrP contains 209 amino acids from residues 23 to 231 with an unstructured N-terminal domain from residues 23 to 127 and a structured C-terminal domain from residues 128 to 231. The 1E4 and 3F4 epitopes are next to each other, localizing in the unstructured domain between residues 97 and 105 for the 1E4 epitope and between residues 106 and 112 for the 3F4 epitope. In the structured C-terminal domain, there are two short β-sheets (PrP128-131 for β1 and PrP161-164 for β2) and three α-helixes (PrP144-154 for α1, PrP173-194 for α2 and PrP200-228 for α3) [26]. There are two N-linked glycosylation sites at residues 181 and 197 (black quad arrow callouts) and a GPI anchor at the residue 231 (black circular arrow) [27, 28].

**Fig. S2**

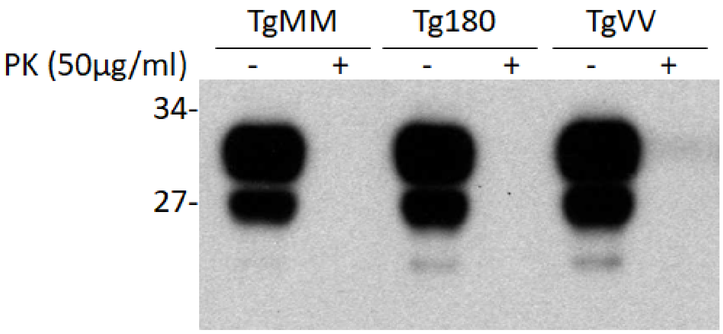


Fig. S2 Comparison of gel profile of the PrP molecule from brain homogenates of humanized TgMM, Tg180 and TgVV mice. Representative Western blotting of PrP from brain homogenates of humanized Tg mice expressing human wild-type PrP with 129MM polymorphism (TgMM), $PrP^{V180I}$ mutation (Tg180), or wild-type PrP with 129VV (TgVV) treated with or without digestion of PK at 50 μg/ml. The blot was probed with 3F4. Molecular weight markers are shown in kDa on the left side of the blots.

Fig. S3

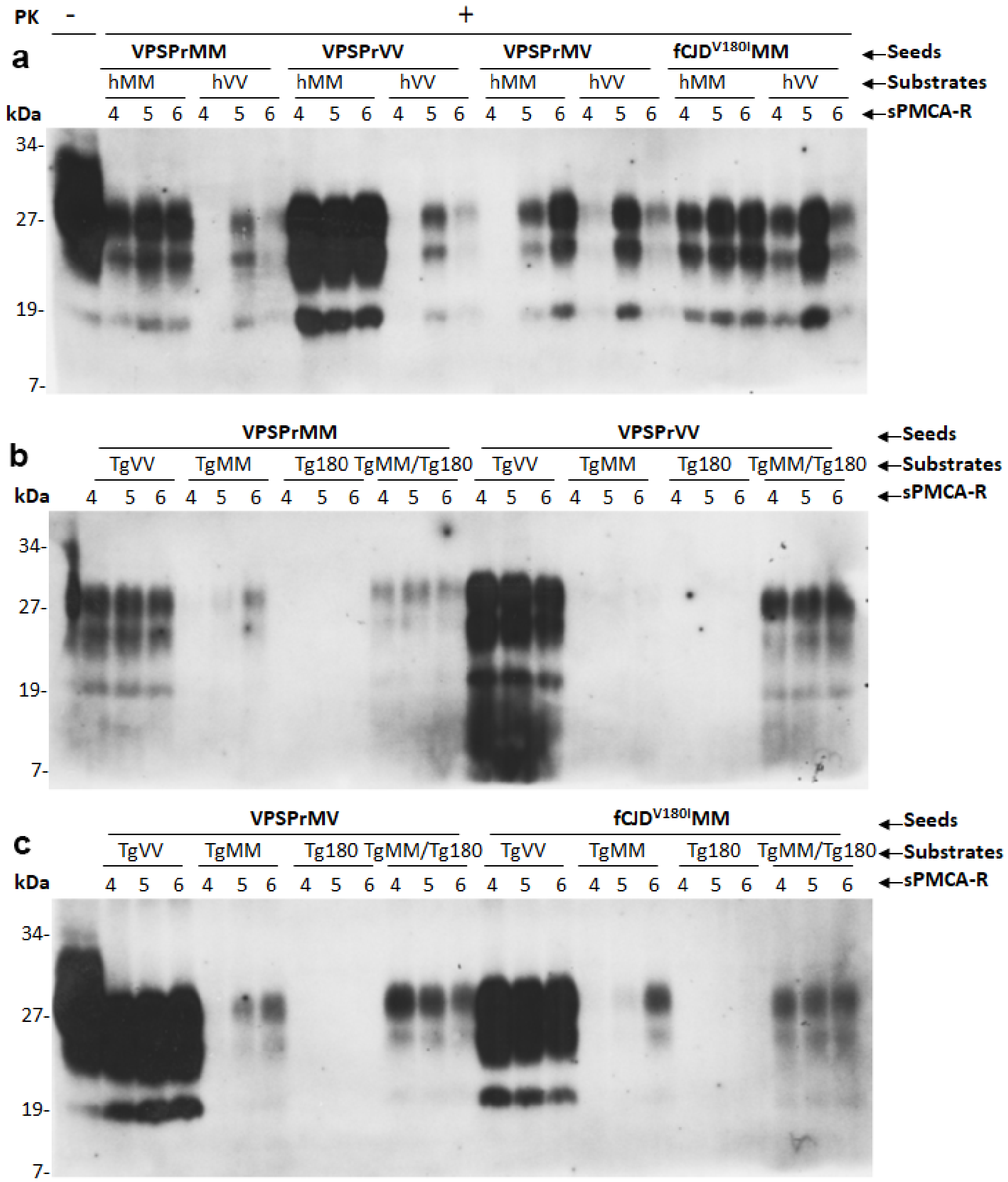


Fig. S3 Serial PMCA of $PrP^{Sc}$ from VPSPr and $fCJD^{V180I}$ in human or Tg mouse brain homogenate substrate. **(a)** Representative Western blotting of $PrP^{Sc}$ amplified with 4-6 rounds of sPMCA by seeding $PrP^{Sc}$ from VPSPrMM, VPSPrVV, VPSPrMV, $fCJD^{V180I}$, and $fCJD^{T183A}$ in the hMM or hVV brain substrate. **(b, c)** Representative Western blotting of $PrP^{Sc}$ amplified with 4-6 rounds of sPMCA by seeding $PrP^{Sc}$ from VPSPrMM and VPSPrVV **(b)** as well as VPSPrMV and $fCJD^{V180I}$MM (**c**) in humanized transgenic mouse brain homogenates from TgVV, TgMM, Tg180, or TgMM + Tg180 mouse lines probed with the

Tohoku 2 antibody. sPMCA-R: sPMCA rounds. Molecular weight markers are shown in kDa on the left side of the blots.

**Fig. S4**

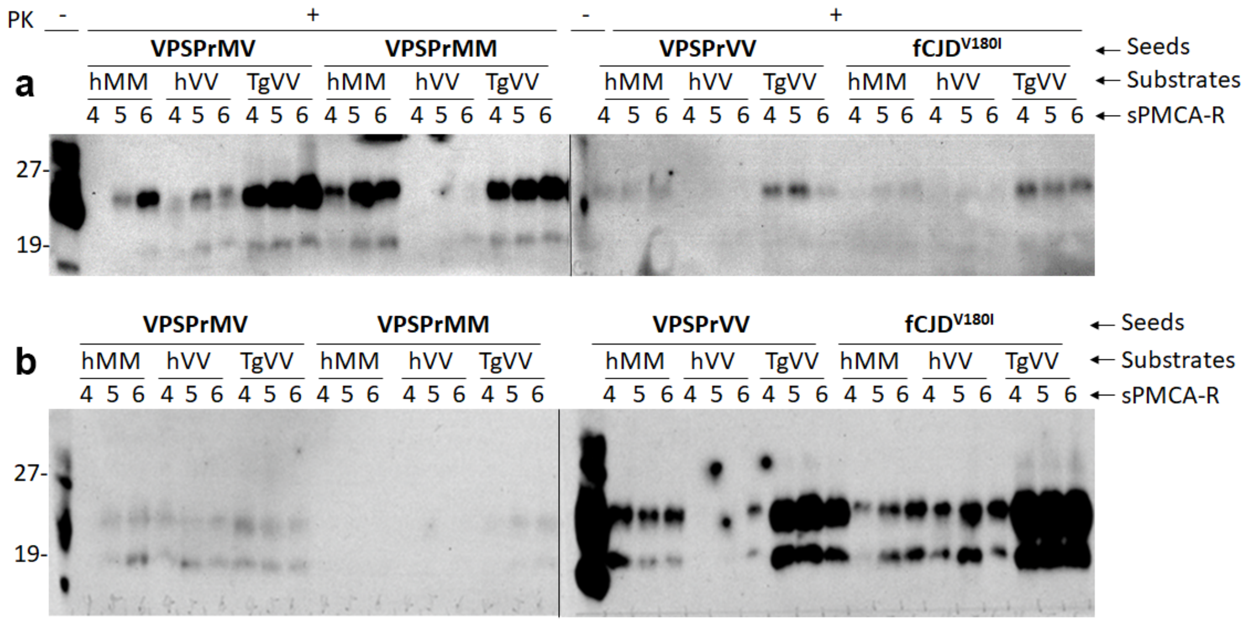


Fig. S4 Serial PMCA of $PrP^{Sc}$ from VPSPr and $fCJD^{V180I}$ in human brain homogenates or humanized Tg mouse brain homogenates. Representative Western blotting of $PrP^{Sc}$ from VPSPrMV, VPSPrMM, VPSPrVV, or $fCJD^{V180I}$ amplified with 4-6 rounds of sPMCA in human brain homogenates from non-CJD MM (hMM) or VV (hVV) or mouse brain homogenates from humanized TgVV mice probed with the Bar209 **(a)** or V14 (**b**) antibody. sPMCA-R: sPMCA rounds. Molecular weight markers are shown in kDa on the left side of the blots. PK: Proteinase K.
